# Sulfatide Deficiency, an Early Alzheimer’s Lipidomic Signature, Causes Brain Ventricular Enlargement in the Absence of Classical Neuropathological Hallmarks

**DOI:** 10.3390/ijms24010233

**Published:** 2022-12-23

**Authors:** Juan Pablo Palavicini, Lin Ding, Meixia Pan, Shulan Qiu, Hu Wang, Qiang Shen, Jeffrey L. Dupree, Xianlin Han

**Affiliations:** 1 Sam and Ann Barshop Institute for Longevity and Aging Studies, University of Texas Health Science Center at San Antonio, San Antonio, TX 78229, USA; 2 Division of Diabetes, Department of Medicine, University of Texas Health Science Center at San Antonio, San Antonio, TX 78229, USA; 3 Department of Biochemistry and Molecular Biology, Soochow University Medical College, Suzhou 215123, China; 4 Research Imaging Institute, University of Texas Health Science Center at San Antonio, San Antonio, TX 78229, USA; 5 Department of Radiology, University of Texas Health Science Center at San Antonio, San Antonio, TX 78229, USA; 6 Department of Anatomy and Neurobiology, Virginia Commonwealth University, Richmond, VA 23284, USA; 7 Research Service, McGuire Veterans Affairs Medical Center, Richmond, VA 23249, USA

**Keywords:** sulfatide, cerebroside sulfotransferase, ventricular enlargement, Alzheimer’s disease, brain MRI, aquaporins

## Abstract

Alzheimer’s disease (AD) is a neurodegenerative disease characterized by progressive memory loss and a decline in activities of daily life. Ventricular enlargement has been associated with worse performance on global cognitive tests and AD. Our previous studies demonstrated that brain sulfatides, myelin-enriched lipids, are dramatically reduced in subjects at the earliest clinically recognizable AD stages via an apolipoprotein E (APOE)-dependent and isoform-specific process. Herein, we provided pre-clinical evidence that sulfatide deficiency is causally associated with brain ventricular enlargement. Specifically, taking advantage of genetic mouse models of global and adult-onset sulfatide deficiency, we demonstrated that sulfatide losses cause ventricular enlargement without significantly affecting hippocampal or whole brain volumes using histological and magnetic resonance imaging approaches. Mild decreases in sulfatide content and mild increases in ventricular areas were also observed in human APOE4 compared to APOE2 knock-in mice. Finally, we provided Western blot and immunofluorescence evidence that aquaporin-4, the most prevalent aquaporin channel in the central nervous system (CNS) that provides fast water transportation and regulates cerebrospinal fluid in the ventricles, is significantly increased under sulfatide-deficient conditions, while other major brain aquaporins (e.g., aquaporin-1) are not altered. In short, we unraveled a novel and causal association between sulfatide deficiency and ventricular enlargement. Finally, we propose putative mechanisms by which sulfatide deficiency may induce ventricular enlargement.

## 1. Introduction

Alzheimer’s disease (AD) is a neurodegenerative disease that is characterized clinically by the progressive loss of memory and other cognitive functions. Clinical and neuropathological studies have greatly advanced our knowledge and revealed that accumulation of amyloid-beta peptide (Aβ) in the brain, which starts decades before symptoms appear, triggers AD pathogenesis, driving neurofibrillary tau pathology, and progressive synaptic, neuronal, and axonal damage [1,2,3]. In addition to neurobiological changes, structural changes in the brain also occur in AD [4,5,6], such as early atrophy of medial temporal structures followed by progressive neocortical damage and ventricular enlargement, which correlate closely with changes in cognitive performance [7,8,9]. Despite decades of intense research, the molecular mechanisms by which Aβ accumulation drives downstream AD neuropathologies are still not fully understood.

Ventriculomegaly is commonly observed in most neurodegenerative disorders, as well as with age [4,5,6,10], and is believed to result from passive enlargement of the lateral, third, and fourth ventricles following brain parenchymal shrinkage [8]. AD subjects demonstrate significantly greater rates of ventricular enlargement compared to both cognitively normal elderly and elderly subjects with mild cognitive impairment (MCI), while elderly MCI subjects display greater rates of enlargement than cognitively normal elderly controls [4,6,11,12,13]. Ventriculomegaly is strongly correlated with a decline in cognitive performance, apolipoprotein E4 (APOE4) genotype, as well as with cerebrospinal fluid and pathologic markers of AD [11,12,13,14]. Finally, larger ventricles in healthy subjects may indicate susceptibility to or progression of dementia-related pathology [4].

Sulfatides are almost exclusively synthesized by oligodendrocytes in the CNS and are present predominantly in the myelin sheath surrounding axons [15,16]. Sulfatides play an important role in oligodendrocyte differentiation and survival, myelin maintenance and function, glial–axon contacts, and proper localization of axonal proteins [17,18,19,20,21,22,23,24]. Altered sulfatide levels in human brain tissues have been associated with the pathogenesis of various human diseases, including metachromatic leukodystrophy, AD, multiple sclerosis, and Parkinson’s disease [15,25,26]. Previously, using a lipidomics approach, we uncovered that brain and cerebrospinal fluid (CSF) sulfatide levels were substantially lost in individuals at the earliest clinically recognizable stages of AD and even at pre-clinical stages [27,28,29]. In addition, we demonstrated that ApoE transports brain sulfatide and modulates its turnover [30]. Specifically, we showed that ApoE particles carry sulfatide in an isoform specific manner, evidenced by the observations that APOE-deficient mice accumulate brain sulfatides and that the brains of transgenic (Tg) mice expressing human APOE4 display reduced sulfatide compared to those of either human APOE3 Tg or wildtype mice [30]. Furthermore, we also demonstrated that APOE is required for Aβ-induced sulfatide deficiency in AD animal models [31] and that adult-onset sulfatide-deficiency leads to AD-like neuroinflammation and cognitive impairment [32,33].

Aquaporins (AQPs) are channel proteins that form pores in the membrane of biological cells, mainly facilitating transport of water between cells [34]. AQP1 and AQP4, the two primary aquaporin molecules of the CNS, regulate brain water and CSF production and movement, controlling the size of intracellular and extracellular fluid volumes, respectively [35]. A simplistic view of their function associates AQP1 with CSF production and AQP4 to CSF/ISF exchange and absorption [36,37].

In this study, we investigated the effects of sulfatide deficiency on brain ventricular size. To this end, we took advantage of sulfatide-deficient mice lacking the gene that encodes the enzyme cerebroside sulfotransferase (CST), which catalyzes the last step of sulfatide biosynthesis [24]. We assessed CST heterozygous (CST^+/−^) mice that display relatively mild reductions of brain sulfatide content [18,38], and to overcome the potential developmental consequences of germline manipulation, we took advantage of a recently generated inducible myelinating cell-specific CST conditional KO (CST cKO) mouse model [33]. To the best of our knowledge, the findings we report here were the first to demonstrate that sulfatide deficiency is sufficient to induce ventricular enlargement, even in the absence of classical AD neuropathological hallmarks, i.e., no amyloid plaques, tau tangles, neuronal death, nor brain shrinkage. Our results have important implications for AD as they suggest that other mechanisms, in addition to brain parenchymal shrinkage, may drive ventricular enlargement. Finally, potential mechanisms leading to ventricular enlargement induced by sulfatide deficiency were investigated.

## 2. Results

### 2.1. Mild Sulfatide Depletion Causes Brain Ventricular Enlargement

**Figure 1:**
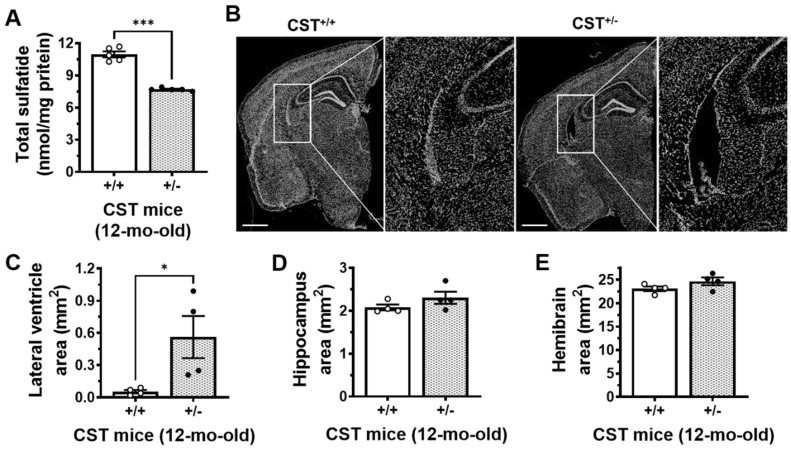
Mild sulfatide deficiency leads to middle-age ventricular enlargement in the mammalian brain. (**A**) Total sulfatide levels in the cerebrum of CST^+/+^ and CST^+/−^ littermate mice measured by MDMS-SL. (**B**) DAPI- (nuclear) stained coronal sections from CST^+/+^ and CST^+/−^ mouse brains on a C57BL/6J background at 12 months of age. Scale bar: 1 mm. Lateral ventricle (**C**), hippocampal (**D**) and whole hemibrain (**E**) areas from CST^+/+^ and CST^+/−^ mice. Anatomical areas were quantified using Image J. N = 4–5 mice/genotype. Each dot represents average data from 2–4 sections (Bregma −1.8 to −2) for each animal. Unpaired two-tailed *t*-test (normality and equal variance were assumed/confirmed). * *p* < 0.05, *** *p* < 0.001.

Multi-dimensional mass spectrometry-based shotgun lipidomics (MDMS-SL) analysis was used to detect sulfatide levels in the cerebrum of CST^+/+^ and CST^+/−^ mice. The results showed that cerebrum sulfatide levels were decreased by approximately 30% (*p* < 0.001) in CST^+/−^ mice compared with CST^+/+^ littermates at 12 months of age (Figure 1A). These results are similar to those previously reported by our group for different brain regions at earlier stages [18,38]. To investigate if sulfatide losses had any effect on brain anatomy, immunohistochemical analysis with DAPI was performed in coronal brain sections of 12-month-old CST^+/+^ and CST^+/−^ mice (Figure 1B). Given the well-established longitudinal changes in hippocampal, lateral ventricle, and brain volume in MCI/AD [8,39,40,41], we decided to focus on these anatomical measurements. Remarkably, mild sulfatide losses led to marked (10-fold, *p* = 0.042) increases in the area of the lateral ventricle (LV) in brain sections at Bregma −1.8 and −2 of CST^+/−^ mice compared to CST^+/+^ controls (Figure 1C). Conversely, no major changes in hippocampal (*p* = 0.204, Figure 1D) or whole brain area (*p* = 0.147, Figure 1E) were observed in CST^+/−^ mice.

**Figure 2:**
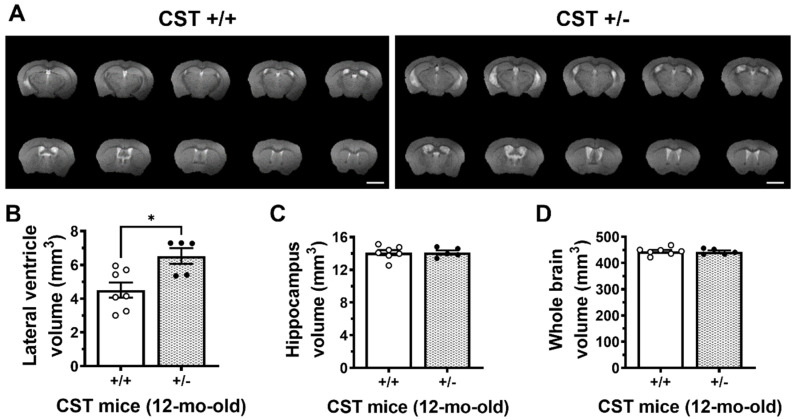
Enlarged brain ventricles in living sulfatide deficient mice. (**A**) Representative MRI images from CST^+/+^ and CST^+/−^ mice. White areas represent water/CSF filled ventricles. Scale bar: 2 mm. Lateral ventricle (**B**), hippocampus (**C**), and whole brain (**D**) volumes were calculated by summing-up the area of each imaged section multiplied by slice thickness for each mouse brain. N = 5–7 mice/genotype. Unpaired two-tailed *t*-test (normality and equal variance were assumed/confirmed). * *p* < 0.05.

Consistently, anatomical brain MRI scans on living mice yielded similar findings (Figure 2A). Specifically, LV volumes were significantly enlarged by 45% (*p* = 0.013) in 12-month-old CST^+/−^ mice compared with CST^+/+^ mice (Figure 2B). Similarly, whole ventricular volume (lateral ventricles + third ventricle + cerebral aqueduct + fourth ventricle) was also increased in sulfatide deficient mice. The increases in ventricular volume occurred across the brain in both rostral and dorsal regions, but were more pronounced in the ventral half of the brain (Bregma −1 to −4). On the other hand, no significant differences between CST^+/−^ and CST^+/+^ mice were detected for hippocampal (*p* = 0.954) or whole brain (*p* = 0.874) volume (Figure 2C,D). Taken together, these results indicate that mild constitutive losses of sulfatides are sufficient to enlarge brain ventricles without affecting brain or hippocampus sizes.

### 2.2. Adult-Onset CNS-Specific Sulfatide Deficiency Leads to Brain Ventricular Enlargement

**Figure 3:**
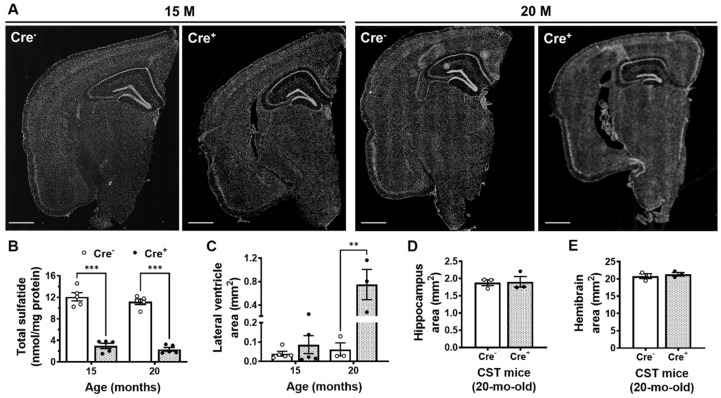
Adult-onset sulfatide deficiency leads to old age ventricular enlargement in the mammalian brain. (**A**) Representative DAPI-stained images of coronal brain sections from 15- and 20-month-old Cre^+^ and Cre^−^ mice following tamoxifen induction at 3 months of age. Scale bar: 1 mm. (**B**) Total sulfatide in cerebrum samples from Cre^+^ and Cre^−^ mice measured by MDMS-SL. Lateral ventricle (**C**), hippocampal (**D**), and whole hemibrain (**E**) areas from mouse brain sections. N = 3–5 mice/genotype. Each dot represents average data from 2–4 sections (Bregma −1.8 to −2) for each animal. Two-way ANOVA (Tukey) for (**A**,**B**) and unpaired two-tailed *t*-test for (**D**,**E**) (normality and equal variance were assumed/confirmed). ** *p* < 0.01, *** *p* < 0.001.

Seeking to overcome the developmental consequences of germline manipulation, we applied a tamoxifen-inducible myelinating glia-specific CST conditional KO mouse model (CST cKO) generated by crossing CST^fl/fl^ and Plp1-CreERT mouse lines as previously described [33]. CST^fl/fl^/Cre^+^ (Cre^+^) and CST^fl/fl^/Plp1-Cre^−^ (Cre^−^) littermate mice were both treated with tamoxifen at 3 months of age, once they reached mature adulthood and myelin had been largely developed [42,43]. Sulfatide levels were dramatically reduced by 75 and 80% in 15- and 20-month-old Cre^+^ mice compared to Cre^−^ littermate controls (*p* < 0.001 for each time point) (Figure 3B). Ventricles in the brains of Cre^+^ mice started trending to increase by 15 months of age and became markedly enlarged (by 12-fold, *p* = 0.002) at 20 months of age compared to Cre^−^ controls (Figure 3A,C). Our results suggest an interaction between sulfatide deficiency and age that seems to drive ventricular enlargement. Finally, consistent with what we observed in CST^+/−^ mice, there were no genotype effects in hippocampal and whole brain areas (*p* = 0.886 and *p* = 0.561, respectively) (Figure 3D,E).

### 2.3. Mild Sulfatide Deficiency and Ventricular Enlargement Observed in APOE4 Compared to APOE2 Knock-in Adult Mice

**Figure 4:**
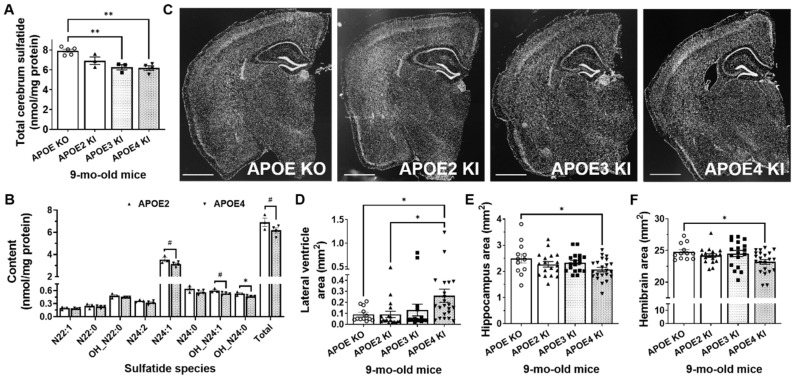
APOE isoform-dependent sulfatide deficiency and brain ventricular enlargement. (**A**) Total sulfatide levels in cerebrum samples from 9-month-old APOE KO, APOE2, APO3, APO4 knock-in (KI) mice were measured by MDMS-SL. (**B**) Individual sulfatide molecular species and total levels in APOE2 and APOE4 KI mouse brains. (**C**) Representative DAPI-stained images of coronal brain sections. Scale bar: 1 mm. Lateral ventricle (**D**), hippocampal (**E**), and whole brain (**F**) areas from mouse brain sections. N = 3–5 mice/genotype. Each dot represents an animal in (**A**) and a brain section (Bregma −1.8 to −2) in (**D**–**F**) (3–5 brain sections/animal). Ordinary one-way ANOVA (Tukey) for (**A**,**E**,**F**); multiple unpaired two-tailed *t*-test for (**B**); Welch ANOVA for (**D**). ^#^ *p* < 0.15, * *p* < 0.05, ** *p* < 0.01.

Cumulative evidence suggests that the ε4 allele of APOE is the major genetic risk factor for Alzheimer’s disease. To further confirm the relationship between sulfatide deficiency and ventricular enlargement, we measured sulfatide levels and ventricular area in human APOE2, APOE3, and APOE4 knock-in mice, as well as in APOE KO mice, at 9 months of age. As expected, compared to APOE3/4-containing brains, APOE KO mouse brains accumulated sulfatide by 26 and 28%, respectively (adj. *p* = 0.003 and adj. *p* = 0.001, respectively). These results are consistent with those previously reported by our group [30]. Interestingly, compared to APOE2-containing brains, APOE KO brains only tended to accumulate sulfatide by 14% (adj. *p* = 0.072) (Figure 4A). Moreover, APOE4 mouse brains tended to have lower levels of total sulfatide content compared to APOE2 mice (−11%, *p* = 0.141) (Figure 4B). Similarly, major sulfatide molecular species were either significantly decreased or tended to decrease in APOE4 compared to APOE2 mice (Figure 4B). Consistent with an association between sulfatide losses and ventricular enlargement, lateral ventricle areas were significantly increased by 2.9-fold (adj. *p* = 0.033) in mice with the lowest sulfatide levels (APOE4 KI) compared to mice with the highest sulfatide levels (APOE KO and APOE2 KI) (Figure 4C,D). Finally, APOE KI mice displayed slight but significant reductions in hippocampal and hemibrain area compared to APOE KO mice (−17% and −6%, adj. *p* = 0.046 and adj. *p* = 0.045, respectively) (Figure 4E,F).

### 2.4. Sulfatide Deficiency Induces Ventricular Enlargement by Increasing AQP4 Expression

**Figure 5:**
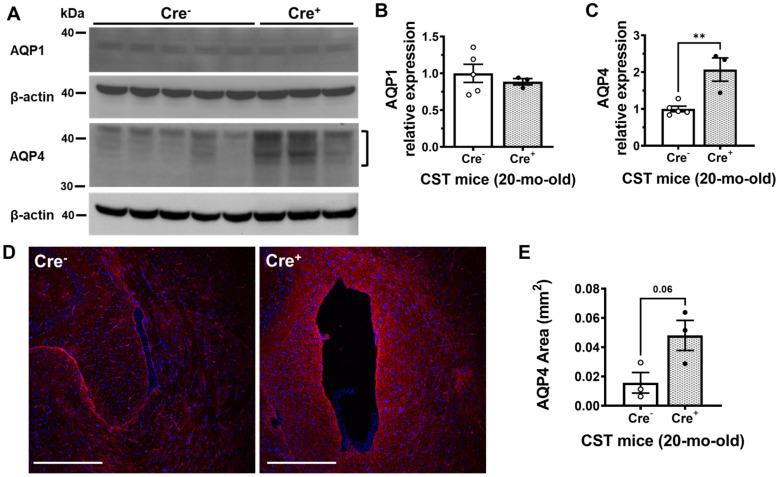
Aquaporin levels in the sulfatide deficient mouse brain. (**A**) Expression of AQP1 and AQP4 in Cre^+^ and Cre^−^ cerebrum samples were measured by Western Blot and quantified (**B**,**C**). Multiple AQP4 bands with different levels of glycosylation were observed (bracket). (**D**) Representative immunofluorescence image for AQP4 (red) in the CST cKO mouse brain at 20 months of age. Scale bar: 250 μm. (**E**) AQP4 immunofluorescent area around the lateral ventricle was quantified using Image J. Unpaired two-tailed *t*-test (normality and equal variance were assumed/confirmed). ** *p* < 0.01.

In an attempt to provide insights into the molecular mechanisms underlying sulfatide-deficiency induced ventricular enlargement, we assessed the two major aquaporin channels in the brain, AQP1 and AQP4. Western blot analysis demonstrated that sulfatide deficiency had no effect on the levels of AQP1 in the brains of Cre^+^ mice compared to Cre^−^ mice (Figure 5A,B). On the other hand, sulfatide deficiency did result in marked ~5-fold increases in brain AQP4 levels (*p* = 0.005) (Figure 5A,C). We validated our biochemical results by immunohistochemistry, where an evident 3-fold increasing trend was observed for AQP4 in the circumventricular region (Figure 5D,E).

## 3. Discussion

Alzheimer’s disease brains are characterized anatomically by enlarged ventricles and biochemically by a significant loss of sulfatide content. In the present study, we found a novel and causal relationship between sulfatide deficiency and ventricular enlargement taking advantage of genetically modified mice. Mice constitutively lacking a copy of the gene that encodes the enzyme that synthesizes sulfatide (CST^+/−^), generate ~70% of the amount of sulfatide found in wild-type littermate controls. Remarkably, this mild decrease in brain sulfatide levels was sufficient to result in a significant increase in ventricular size by middle age (12 months). Adult onset sulfatide losses in CST cKO mice also led to ventricular enlargements, which became most dramatic by old age (20 months). The temporal pattern of ventricular enlargement observed as sulfatide-deficient CST cKO aged, was analogous to the progressive increase in ventricular size described with normal aging in humans, where gradual progressive increases in ventricular size during the first sixth decades are followed by exponential increases in the eighth and ninth decades [10]. As sulfatide deficiency has also been described with age in both mice [30,44] and humans [45,46,47], we speculate that sulfatide deficiency may contribute to ventricular enlargement not only in AD but also in normal aging.

Previously, we demonstrated that apolipoprotein E mediates sulfatide depletion in animal models of Alzheimer’s disease [31], and that sulfatide levels in brain tissue from middle-age APOE4-expressing transgenic mice were lower than those found in wild-type mice [30]. In the current study, APOE3 and APOE4 KI mice displayed lower sulfatide levels compared to APOE KO mice. Consistent with the decreases in total brain sulfatides, ventricular sizes increased in APOE4 compared to APOE2 KI or APOE KO mice. It seems reasonable to speculate that the mild differences in brain sulfatide content and ventricular size observed during mature adulthood between APOE genotypes are likely to become more dramatic with advanced age.

Enlargement of the ventricles may occur for a number of reasons, potential explanations include loss of brain volume, impaired production, outflow, or absorption of cerebrospinal fluid from the ventricles [48]. In the present study, we provide evidence that sulfatide depletion leads to ventricular enlargement in the absence of brain volume or hippocampal shrinkage, suggesting that this effect is independent of major brain atrophy or neuronal death. Supporting this notion, our group has previously reported that sulfatide deficient mice do not exhibit major neuronal loss [33]. In fact, CST constitutive and conditional KO mouse brain sections did not display increased DNA fragmentation/apoptosis in the cortex or hippocampus following TUNEL staining [33]. Consistently, cell type specific NanoString scores (a proxy for neuronal and glial numbers) were not significantly altered for neurons, and were slightly increased for astrocytes, microglia, and oligodendrocytes in sulfatide depleted and/or deficient mice [33]. Taken together, our data largely rules out the possibility that the ventricular enlargement induced by sulfatide deficiency is due to reductions in brain cell numbers.

Interestingly, we found that brain AQP4 levels were significantly increased in CST cKO mice under extreme sulfatide deficient conditions. Single-cell/nuclei RNA sequencing studies have consistently revealed that AQP4 is primarily expressed by astrocytes in the mouse and human brain [49,50,51,52]. Considering that we have previously shown that sulfatide losses lead to reactive astrogliosis [33], we speculate that the observed increases in AQP4 may be driven by astrocyte activation. Notably, proteomic data from post-mortem brains of more than 500 individuals from Agora, a platform initially developed by the NIA-funded AMP-AD consortium that shares evidence in support of AD target discovery, has revealed that AD brains (dorsolateral prefrontal cortex) display significantly higher levels of AQP4 compared to controls (https://agora.adknowledgeportal.org/, accessed date on 13 September 2022). As mentioned earlier, expression of AQP4 is particularly high around areas in contact with cerebrospinal fluid, suggesting that AQP4 plays a role in fluid exchange between the cerebrospinal fluid compartments and the brain [53]. AQP4 channels have been reported to increase after two weeks of hydrocephalus to facilitate CSF absorption from the ventricle into the parenchymal extracellular space [54]. Given that AQP4 null mice show accelerated progression of ventriculomegaly relative to wild type controls [55], increases in cerebral AQP4 have been proposed to be neuroprotective [54]. Thus, the increase in AQP4 expression under sulfatide deficient conditions presented here, might represent an adaptive response to increases in CSF, i.e., hydrocephalus. Supporting this notion, AQP4 increases were only significantly increased in CST cKO at the latest time point analyzed and were not significantly altered in CST^+/−^ mice.

Alternatively, the ventricular system may be dilated under sulfatide deficient conditions as a result of an abnormal accumulation of CSF due to defects in CSF circulation. Supporting this notion, while collecting CSF from anesthetized CST cKO mice, our group has noticed that Cre^+^ mice typically yield larger CSF volumes than Cre^−^ mice. Consistent with this idea, deep white matter ischemia during late adulthood, characterized histologically by myelin pallor (i.e., loss of lipid), has been shown to result in hydrocephalus [56]. Similarly, the attraction between the bare myelin protein and the CSF has been reported to increase resistance to the extracellular outflow of CSF, causing it to back up, resulting in hydrocephalus [56]. Notably, we have shown that sulfatide deficient mice display substantial decreases of virtually all myelin-enriched lipids and a reduction in several major myelin proteins [18], both of which could impair proper CSF flow. Finally, considering that the sulfate group of sulfatides confers a negative charge and that sulfatides are the major negatively charged lipid in the outer leaflet of the myelin membrane, sulfatide losses may also result in a significant disruption of charge balance in the brain, particularly in the interplay between myelin-extracellular space, where interstitial fluid (ISF) circulates. Thus, sulfatide losses could potentially also impact the flow of ISF and the contiguous CSF by disrupting charge balance. Taken together our data led us to propose that the observed ventricular enlargement in sulfatide deficient mouse brains is not driven by neurodegeneration but rather by increased resistance to the outflow of CSF that eventually lead to an abnormal buildup of fluid, i.e., hydrocephalus.

Finally, it seems reasonable to speculate that enlarged ventricles in sulfatide deficient mice will be associated with cognitive dysfunction. In fact, we recently reported evidence of impaired spatial and non-spatial recognition memory in sulfatide deficient mice [33], at a time point where we know thanks to the current study that ventricles are just starting to display increasing trends. Unfortunately, sulfatide deficient mice also display locomotor defects that aggravate as sulfatide levels decrease and ventricles enlarge. Given that locomotor abnormalities complicate and confound cognitive studies, dissecting the relationship between ventricular enlargement and cognition in sulfatide deficient mice becomes technically challenging. Future electrophysiological studies (e.g., long-term potentiation recordings) on sulfatide deficient mice could provide valuable insights into the relationship between ventricular enlargement and learning and memory.

In summary, our present study revealed a novel and causal association between sulfatide deficiency and ventricular enlargement; and provided insights into the putative mechanisms by which sulfatide deficiency may disrupt CSF circulation resulting in an accumulation of CSF that drives hydrocephalus. These results, together with previous studies from our group and others, suggest that preventing or restoring brain sulfatide deficiency may be an effective strategy to treat/delay AD and brain aging.

## 4. Materials and Methods

### 4.1. Mice

CST^+/−^ mice, originally obtained from our collaborator Dr. Jeffery Dupree, have been housed and bred in our laboratory for more than a decade. CST^+/−^ have been backcrossed with C57BL/6J mice multiple times. CST^+/−^ and CST^+/+^ littermate control mice (N = 4–7 mice/group including males and females) were assessed at 12 months of age. CST^fl/fl^ mice, generated by Dr. Dupree, were crossed with Plp1-Cre/ERT^+^ mice (Stock No: 005975, the Jackson Laboratory) as previously described [33]. Tamoxifen (40 mg tamoxifen/kg body weight) was administered via intraperitoneal injection daily for a total of 4 consecutive days to CST^fl/fl^/Plp1-CreERT^−^ (Cre^−^) and CST^fl/fl^/Plp1-CreERT^+^ (Cre^+^) littermate mice at 3 months of age. Female Cre^−^ and Cre^+^ littermates were assessed at 15 and 20 months of age (N = 3–5 mice/group). Human APOE2, APOE3 and APOE4 knock-in C57BL/6 mice (N = 4–5 males/group) were purchased from the Taconic Biosciences and assessed at 9-months of age.

All mice were housed in groups of ≤5 mice/cage, maintained in a temperature- and humidity-controlled environment with a 12 h light–dark cycle, and provided with food and water ad libitum. The protocols for animal experiments were conducted in accordance with the ‘Guide for the Care and Use of Laboratory Animals (8th edition, National Research Council of the National Academies, 2011) and were approved by the Animal Studies Committee of the University of Texas Health Science Center at San Antonio.

### 4.2. Histology

Mouse brains were dissected, fixed in 4% paraformaldehyde, cryoprotected with sucrose 10–30%, embedded in optimal cutting temperature compound (OCT), and frozen. Cryostat brain 10 μm-thick sections were mounted on positively charged slides (Fisherbrand Supefrost Plus). Two to four sections for each animal between Bregma −1.8 and −2 were averaged and used for quantification. For ventricular analysis, PBS-rinsed slides were directly added 4′,6-diamidino-2-phenylindole (DAPI)-containing mounting media (Vectashield; Vector Laboratories, Burlingame, CA, USA). Brain sections from 20 months-old CST cKO mice were incubated with anti-AQP4 (NBP1-87679, Novus, St Louis, MO, USA) overnight at 4 °C and incubated with secondary Alexa Fluor 568 antibody (A-11036, Invitrogen, Waltham, MA, USA) for 1 h at room temperature (~23 °C) followed by addition of DAPI-containing mounting media. Images were taken using a KEYENCE fluorescence microscope (BZ-X800) and analyzed using BZ-800 analyzer and ImageJ software.

### 4.3. Lipidomics

Briefly, fresh and/or frozen cerebral tissue was homogenized in ice-cold 0.1 × phosphate-buffered saline (PBS) using Precellys^®^ Evolution Tissue Homogenizer (Bertin, France) as previously described [18]. Protein concentration of homogenates was determined using the bicinchoninic acid protein assay (Thermo Fisher Scientific, New York, NY, USA). Lipids were extracted by a modified procedure of Bligh and Dyer extraction in the presence of internal standards, which were added based on the total protein content of each sample [57,58,59]. Lipids were assessed using a triple-quadrupole mass spectrometer (TSQ Altis (Thermo Fisher Scientific, Waltham, MA, USA) equipped with a Nanomate device (Advion, Ithaca, NY, USA) and Xcalibur system as previously described [60,61,62]. Data processing including ion peak selection, baseline correction, data transfer, peak intensity comparison, ^13^C deisotoping, and quantitation were conducted using a custom programmed Microsoft Excel macro as previously described after considering the principles of lipidomics [61,62].

### 4.4. Brain MRI

Magnetic resonance imaging (MRI) experiments were performed on an 11.7 Tesla scanner (BioSpec, Bruker, Billerica, MA, USA). A surface coil was used for brain imaging. Mice were maintained on 1.5% isoflurane anesthesia while imaged. Anatomical images were obtained using a fast spin-echo sequence with a matrix = 128 × 128, field of view (FOV) = 1.28 cm × 1.28 cm, repetition time (TR) = 4000 ms, and effective echo time = 25 ms. Thirty 1 mm coronal images were acquired with six averages. Total scan time was ~12 min. MRI analysis was conducted using Stimulate software [63] ran on a CentOS5 Linux Operating System. Anatomical MRI images were used to measure whole brain, hippocampi, and ventricular volume. Whole brain volume was obtained by ROI trace after removal of the skull using a local Gaussian distribution 3D segmentation MATLAB code [64]. The desired regions of interest (ROIs) were outlined manually for right and left hippocampi. Ventricular volume was obtained by thresholding anatomical image voxels (by setting up an appropriate floor value for each animal) to highlight regions of greater intensity, followed by ROI defining the target regions (excluding relatively rare non-specific highlighted voxels). Volumes were obtained by multiplying ROI total voxels (hot points) by voxel volume (0.004 mm^3^).

### 4.5. Western Blot

Cerebrum tissue was homogenized in NP40 buffer with Halt Protease and Phosphatase Inhibitor Cocktails (Thermo Fisher Scientific, Waltham, MA, USA) using Precellys^®^ Evolution Tissue Homogenizer (Bertin, Technologies, Montigny-le-Bretonneux, France). Homogenates were centrifuged at 12,000× *g* for 30 min at 4 °C. Supernatants (30 µg protein) were run with NuPage 4–12% Bis-Tris gels (Thermo Fisher Scientific, Waltham, MA, USA) under reducing conditions. Western blot analyses were performed using antibodies against AQP1 (ab65837, Abcam, Waltham, MA, USA), AQP4 (NBP1-87679, Novus, USA), and β-actin (7074P2, Cell Signaling Technology, Danvers, MA, USA). Relative intensities were quantified using ImageJ software and normalized to β-actin.

### 4.6. Statistical Analysis

The results were presented as mean ± standard error of the mean (SEM). All data were subjected to analysis of variance using GraphPad Prism software (version 9). Comparisons of two groups were performed using two-tailed unpaired *t*-tests. When appropriate, multiple comparisons between groups were assessed via one-way ordinary or Welch (if datasets did not pass normality tests and were heteroscedastic) ANOVA, or two-way ANOVA, followed by Tukey (ordinary) or Benjamini, Krieger and Yekutieli (Welch) post hoc tests. * *p* < 0.05, ** *p* < 0.01, *** *p* < 0.001.

## Data Availability

Not applicable.
